# Supplementary material for: Decline of Common Toad Populations in Flanders Is Not Linked to Surrounding Landscape
Source: Ecol Evol. 2026 May 17;16(5):e73653. doi: 10.1002/ece3.73653 (PMC13180481; doi:10.1002/ece3.73653)

**Appendix:**

Appendix Table S1:

This Table gives the output for each population with a time series longer than 5 years. For each toad patrol, there is a unique location number, street name and province. The adjusted p values from the omnibus test and the post-hoc test are shown and together with the trend value, the status of the toad population (label; D = decrease, I = increase, F = flexible and NS = non-significant) can be assigned to the population. When the omnibus test is non-significant (omnibus p_adj_ > 0.05), the trend of the population is non-significant. Otherwise there is a trend, which is either flexible (p_adj_ > 0.05), increasing or decreasing, depending on the sign of the trend value. Furthermore, the kind of model that is used to estimate the trend of the population (GAM = Generalized Additive Model; GLM = Generalized Linear Model), amount of observations (# obs) and the timeframe (start-end) within they are observed are given. When the trend is estimated with a GLM, there is no omnibus p-value, which results that these sites cannot have flexible as label.

| **Site id** | **Street name** | **Province** | **Omnibus p_adj_** | **p_adj_** | **Trend value** | **Label** | **Model** | **Start** | **End** | **# obs** |
| --- | --- | --- | --- | --- | --- | --- | --- | --- | --- | --- |
| 5 | Abdij Van Park | Vlaams Brabant | 1.66E-02 | 1.11E-03 | -0.27 | D | GAM | 2010 | 2021 | 13 |
| 10 | Aertschouw (Opoeteren) | Limburg | 9.19E-01 |  | 0.01 | NS | GAM | 2011 | 2022 | 12 |
| 15 | Appeldijkstraat (Weert) | Antwerpen | 2.61E-03 | 2.81E-02 | -0.06 | D | GAM | 1994 | 2007 | 11 |
| 16 | Arendsnest | Antwerpen | 2.90E-05 | 2.88E-10 | 0.15 | I | GAM | 2009 | 2022 | 12 |
| 25 | Ballewijerweg | Limburg | 6.87E-03 | 1.91E-03 | -0.05 | D | GAM | 1988 | 2022 | 17 |
| 27 | Bankelindeweg | West-Vlaanderen | 2.18E-06 | 2.45E-02 | 0.03 | I | GAM | 2000 | 2022 | 18 |
| 32 | Beelbroekstraat | Oost-Vlaanderen | 1.95E-05 | 0.00E+00 | -0.48 | D | GAM | 2013 | 2022 | 10 |
| 41 | Bergstraat (Kemmel) | West-Vlaanderen | 3.42E-01 |  | 0.00 | NS | GAM | 1988 | 2021 | 30 |
| 43 | Bergstraat | Antwerpen | 4.68E-05 | 9.82E-02 | -0.03 | F | GAM | 1993 | 2022 | 25 |
| 45 | Berlaarbaan | Antwerpen | 2.53E-02 | 5.89E-04 | -0.15 | D | GAM | 2007 | 2022 | 10 |
| 60 | Bleukstraat | Antwerpen | 3.14E-04 | 2.81E-10 | -0.26 | D | GAM | 2011 | 2022 | 11 |
| 61 | Blinckaertduinbos | West-Vlaanderen | 3.55E-03 | 6.76E-01 | 0.02 | F | GAM | 2007 | 2022 | 10 |
| 67 | Borggravevijverstraat | Limburg | 7.20E-04 | 2.30E-03 | -0.04 | D | GAM | 1988 | 2017 | 17 |
| 79 | Bramensdam (Bazel) | Oost-Vlaanderen | 6.31E-03 | 2.14E-06 | 0.14 | I | GAM | 2002 | 2022 | 13 |
| 86 | Buitenland | Antwerpen | 1.61E-03 | 2.17E-08 | -0.13 | D | GAM | 2013 | 2022 | 10 |
| 89 | Burchtstraat (Kolmont) | Limburg | 1.73E-02 | 1.03E-01 | -0.02 | F | GAM | 1989 | 2022 | 11 |
| 91 | Bussegem | Oost-Vlaanderen | 3.42E-01 |  | 0.00 | NS | GAM | 2012 | 2022 | 11 |
| 100 | Damstraat (Opdorp) | Oost-Vlaanderen | 9.34E-09 | 0.00E+00 | -0.17 | D | GAM | 2011 | 2022 | 12 |
| 105 | De Pont | Antwerpen | 4.54E-02 | 1.00E+00 | 0.00 | F | GAM | 2006 | 2022 | 17 |
| 106 | De Weert | Antwerpen | 2.77E-03 | 2.37E-09 | -0.13 | D | GAM | 2013 | 2022 | 10 |
| 131 | Eesbeekstraat | Oost-Vlaanderen | 1.59E-01 |  | -0.14 | NS | GAM | 2009 | 2020 | 11 |
| 136 | Eindepoel | Antwerpen | 2.49E-03 | 2.22E-05 | -0.07 | D | GAM | 2012 | 2022 | 12 |
| 138 | Einhovensebaan | Antwerpen | 1.19E-01 |  | -0.04 | NS | GAM | 2004 | 2022 | 15 |
| 152 | Fietsostrade Lint - Lier | Antwerpen | 1.33E-01 |  | -0.06 | NS | GAM | 2013 | 2022 | 10 |
| 156 | Fort 8 | Antwerpen | 4.38E-03 | 1.00E+00 | 0.01 | F | GAM | 2011 | 2022 | 12 |
| 163 | Ganzendam (Vurste) | Oost-Vlaanderen | 2.76E-02 | 4.90E-04 | -0.11 | D | GAM | 2011 | 2022 | 12 |
| 165 | Gaverbosdreef, S. Van De Veldestraat | Oost-Vlaanderen | 2.69E-02 | 9.28E-03 | -0.19 | D | GAM | 2013 | 2022 | 10 |
| 200 | Heiken | Antwerpen | 2.16E-01 |  | 0.00 | NS | GAM | 1986 | 2022 | 23 |
| 202 | Heirbaan | Vlaams Brabant | 7.97E-03 | 1.67E-03 | -0.25 | D | GAM | 2013 | 2022 | 10 |
| 209 | Hobokense Polder | Antwerpen | 2.28E-01 |  | -0.02 | NS | GAM | 1996 | 2022 | 19 |
| 211 | Hoekstraat (Heppen) | Limburg | 4.89E-02 | 4.93E-02 | -0.04 | D | GAM | 2002 | 2022 | 21 |
| 215 | Hof Ter Bollendreef (Liezele) | Antwerpen | 3.46E-03 | 3.23E-04 | -0.08 | D | GAM | 1989 | 2019 | 16 |
| 227 | Honegemstraat | Oost-Vlaanderen | 2.62E-01 |  | -0.05 | NS | GAM | 2012 | 2022 | 10 |
| 232 | Hospitaalstraat (Vlamertinge) | West-Vlaanderen | 2.63E-01 |  | 0.01 | NS | GAM | 1988 | 2022 | 12 |
| 245 | Kasteeldreef, Bloemenlei | Antwerpen | 9.09E-11 | 7.49E-13 | -0.18 | D | GAM | 2010 | 2021 | 12 |
| 247 | Kasteelhoekstraat (Hollebeke) | West-Vlaanderen | 2.47E-02 | 1.97E-02 | -0.05 | D | GAM | 1998 | 2022 | 11 |
| 255 | Kattenbroek | Antwerpen | 9.71E-03 | 1.03E-01 | -0.14 | F | GAM | 2009 | 2021 | 11 |
| 258 | Kerkedreef | Antwerpen | 3.83E-03 | 2.90E-03 | -0.13 | D | GAM | 1993 | 2021 | 12 |
| 272 | Kloosterbeekstraat | Limburg | 4.03E-02 | 2.10E-02 | 0.08 | I | GAM | 2003 | 2017 | 12 |
| 273 | Kluisbaan | Antwerpen | 3.60E-03 | 4.19E-04 | -0.16 | D | GAM | 2009 | 2022 | 11 |
| 280 | Kokerellestraat | Oost-Vlaanderen | 4.41E-04 | 4.91E-07 | -0.15 | D | GAM | 2013 | 2022 | 10 |
| 295 | Kruineikestraat | Vlaams Brabant | 2.61E-01 |  | -0.06 | NS | GAM | 2013 | 2022 | 10 |
| 300 | Kwarikweg | Oost-Vlaanderen | 1.59E-03 | 2.94E-01 | -0.02 | F | GAM | 2003 | 2021 | 13 |
| 318 | Lichtaartseweg | Antwerpen | 2.73E-02 | 2.73E-02 | -0.06 | D | GAM | 1998 | 2022 | 10 |
| 319 | Liedermeersweg | Oost-Vlaanderen | 4.74E-05 | 2.50E-05 | -0.04 | D | GAM | 1991 | 2022 | 26 |
| 331 | Lusthoflaan (Wondelgem) | Oost-Vlaanderen | 6.21E-02 |  | 0.07 | NS | GAM | 2012 | 2022 | 11 |
| 334 | Makkegemstraat (Schelderode) | Oost-Vlaanderen | 4.68E-05 | 4.84E-04 | -0.09 | D | GAM | 2001 | 2022 | 22 |
| 344 | Meierij (Schelderode) | Oost-Vlaanderen | 3.42E-01 |  | -0.02 | NS | GAM | 2011 | 2021 | 11 |
| 346 | Meirestraat | Oost-Vlaanderen | 5.91E-03 | 2.80E-04 | -0.10 | D | GAM | 2009 | 2021 | 12 |
| 347 | Merbeekstraat | Vlaams Brabant | 2.47E-02 | 7.40E-01 | 0.02 | F | GAM | 2007 | 2022 | 15 |
| 348 | Mereldreef | Vlaams Brabant | 2.55E-01 |  | 0.05 | NS | GAM | 2011 | 2020 | 10 |
| 351 | Middelberg | Vlaams Brabant | 1.42E-03 | 1.69E-05 | -0.12 | D | GAM | 2012 | 2022 | 11 |
| 353 | Mikse Baan 1 | Antwerpen | 5.31E-03 | 3.65E-04 | -0.12 | D | GAM | 2011 | 2022 | 12 |
| 354 | Mikse Baan 2 | Antwerpen | 7.63E-07 | 0.00E+00 | -0.24 | D | GAM | 2011 | 2022 | 12 |
| 360 | Molenlei | Antwerpen | 5.85E-02 |  | -0.06 | NS | GAM | 2011 | 2022 | 11 |
| 361 | Molenschansweg | Limburg | 2.49E-03 | 3.30E-01 | -0.01 | F | GAM | 1988 | 2022 | 14 |
| 367 | Moorselestraat | West-Vlaanderen | 1.00E-01 |  | -0.25 | NS | GAM | 2012 | 2021 | 10 |
| 374 | Nachtegaalstraat | West-Vlaanderen | 4.20E-01 |  | -0.02 | NS | GAM | 2003 | 2022 | 18 |
| 384 | Nijlensesteenweg | Antwerpen | 1.59E-01 |  | 0.01 | NS | GAM | 1998 | 2022 | 14 |
| 386 | Normandiestraat | West-Vlaanderen | 5.44E-04 | 2.86E-11 | -0.13 | D | GAM | 2011 | 2022 | 12 |
| 396 | Opstraat | Limburg | 2.49E-03 | 4.26E-04 | 0.07 | I | GAM | 1995 | 2022 | 12 |
| 398 | Oude Galgenstraat | Antwerpen | 1.24E-04 | 8.53E-03 | 0.06 | I | GAM | 2001 | 2021 | 13 |
| 401 | Oude Maasstraat | Limburg | 9.73E-02 |  | -0.05 | NS | GAM | 2001 | 2021 | 12 |
| 403 | Oude Schansstraat (Zelem) | Limburg | 1.44E-02 | 8.13E-03 | -0.03 | D | GAM | 1994 | 2022 | 25 |
| 404 | Oude Scheldestraat (Kaaihoeve) | Oost-Vlaanderen | 9.76E-08 | 0.00E+00 | -0.19 | D | GAM | 2013 | 2022 | 10 |
| 410 | Paddenbroek | Oost-Vlaanderen | 3.36E-02 | 1.66E-02 | -0.04 | D | GAM | 1996 | 2022 | 13 |
| 414 | Palokenstraat | Vlaams Brabant | 3.33E-04 | 2.22E-07 | 0.18 | I | GAM | 2007 | 2022 | 14 |
| 415 | Pannehuisstraat | Limburg | 7.10E-02 |  | -0.02 | NS | GAM | 2001 | 2022 | 16 |
| 418 | Pardasssenhoek | Oost-Vlaanderen | 5.25E-02 |  | -0.12 | NS | GAM | 2011 | 2021 | 10 |
| 426 | Peerlaarstraat | Antwerpen | 1.26E-02 | 1.00E+00 | 0.01 | F | GAM | 2013 | 2022 | 10 |
| 428 | Perreveld | Vlaams Brabant | 3.32E-03 | 6.05E-05 | -0.12 | D | GAM | 2001 | 2022 | 16 |
| 431 | Pierlapont (Loppem) | West-Vlaanderen | 1.19E-08 | 0.00E+00 | -0.23 | D | GAM | 2012 | 2022 | 11 |
| 450 | Reitstraat (Helchteren) | Limburg | 4.68E-05 | 2.72E-08 | -0.09 | D | GAM | 2009 | 2019 | 10 |
| 455 | Reukenstraat | Vlaams Brabant | 1.43E-05 | 0.00E+00 | -0.42 | D | GAM | 2013 | 2022 | 10 |
| 457 | Rhodesgoed (Kachtem) | West-Vlaanderen | 1.11E-06 | 0.00E+00 | -0.22 | D | GAM | 2011 | 2022 | 12 |
| 462 | Rode Dreef | Antwerpen | 5.77E-02 |  | -0.09 | NS | GAM | 2011 | 2022 | 12 |
| 467 | Romeinse Kassei (Voort) | Limburg | 5.38E-06 | 2.53E-15 | -0.16 | D | GAM | 2012 | 2022 | 11 |
| 474 | Rubenskasteel (Weerde) | Vlaams Brabant | 1.08E-01 |  | -0.05 | NS | GAM | 2011 | 2022 | 12 |
| 480 | Schaapstraat | Antwerpen | 4.20E-05 | 4.39E-09 | -0.11 | D | GAM | 2006 | 2022 | 14 |
| 488 | Schilder Evenepoelstraat | Vlaams Brabant | 8.14E-05 | 1.28E-08 | -0.10 | D | GAM | 2007 | 2021 | 11 |
| 489 | Schipdonkbrug | Oost-Vlaanderen | 6.38E-05 | 2.63E-09 | -0.18 | D | GAM | 2008 | 2022 | 14 |
| 492 | Schooldreef | Antwerpen | 4.05E-04 | 5.90E-01 | 0.02 | F | GAM | 2011 | 2022 | 12 |
| 494 | Schoonberg | Oost-Vlaanderen | 3.10E-01 |  | 0.04 | NS | GAM | 2013 | 2022 | 10 |
| 498 | Scouselestraat (Temse) | Oost-Vlaanderen | 2.96E-04 | 3.12E-02 | 0.07 | I | GAM | 2008 | 2022 | 13 |
| 501 | Senthout | Antwerpen | 4.68E-05 | 1.35E-02 | 0.04 | I | GAM | 1994 | 2022 | 23 |
| 516 | Smesstraat | Oost-Vlaanderen | 8.31E-02 |  | 0.09 | NS | GAM | 2011 | 2022 | 11 |
| 518 | Spekstraat (Hallaar) | Antwerpen | 6.19E-01 |  | -0.01 | NS | GAM | 1998 | 2022 | 22 |
| 519 | Spichtstraat | Vlaams Brabant | 2.85E-04 | 8.83E-11 | -0.16 | D | GAM | 2006 | 2022 | 10 |
| 529 | St-Geertruistraat (Neerreppen) | Limburg | 7.46E-03 | 8.09E-03 | -0.08 | D | GAM | 2006 | 2022 | 13 |
| 530 | St-Pietersstraat | West-Vlaanderen | 8.01E-02 |  | -0.05 | NS | GAM | 1998 | 2022 | 11 |
| 532 | Steenberg | Oost-Vlaanderen | 5.77E-02 |  | -0.03 | NS | GAM | 1999 | 2020 | 12 |
| 534 | Steenstortstraat (Beverlo) | Limburg | 1.82E-03 | 7.45E-05 | -0.08 | D | GAM | 2006 | 2021 | 10 |
| 536 | Steenweg Wijchmaal | Limburg | 5.21E-05 | 3.81E-03 | 0.05 | I | GAM | 2004 | 2022 | 18 |
| 542 | Sulferberg (Westouter) | West-Vlaanderen | 2.77E-03 | 1.55E-06 | 0.22 | I | GAM | 1993 | 2003 | 10 |
| 552 | Toekomstlaan | Antwerpen | 4.68E-05 | 4.51E-03 | -0.03 | D | GAM | 1989 | 2019 | 22 |
| 568 | Vijverstraat | Limburg | 2.96E-02 | 9.71E-02 | -0.04 | F | GAM | 2006 | 2022 | 14 |
| 574 | Voordestraat (Humbeek) | Vlaams Brabant | 3.42E-01 |  | -0.01 | NS | GAM | 2010 | 2022 | 13 |
| 576 | Voort | Antwerpen | 2.49E-03 | 5.34E-05 | -0.14 | D | GAM | 2011 | 2022 | 12 |
| 577 | Vosberg | Antwerpen | 4.54E-02 | 6.50E-01 | -0.02 | F | GAM | 2011 | 2022 | 12 |
| 580 | Vroegeinde | Antwerpen | 2.91E-04 | 4.56E-08 | -0.15 | D | GAM | 2011 | 2022 | 12 |
| 585 | Waasmunsterbaan | Oost-Vlaanderen | 9.34E-09 | 1.86E-10 | -0.06 | D | GAM | 1988 | 2022 | 26 |
| 587 | Wallemote/Wolvenhof | West-Vlaanderen | 1.24E-01 |  | 0.05 | NS | GAM | 2011 | 2021 | 11 |
| 597 | Weehaagstraat (Eksaarde) | Oost-Vlaanderen | 5.26E-05 | 0.00E+00 | -0.40 | D | GAM | 2006 | 2017 | 11 |
| 602 | Wijk Monteval | West-Vlaanderen | 3.76E-02 | 1.33E-01 | -0.10 | F | GAM | 2011 | 2022 | 10 |
| 606 | Wilderhof | Vlaams Brabant | 9.09E-11 | 2.53E-15 | -0.32 | D | GAM | 2009 | 2022 | 11 |
| 607 | Wildersedijk | Antwerpen | 6.71E-01 |  | 0.01 | NS | GAM | 1998 | 2022 | 16 |
| 608 | Wilgenbroekstraat | West-Vlaanderen | 2.49E-03 | 3.74E-01 | -0.05 | F | GAM | 1991 | 2003 | 12 |
| 610 | Witte Bomendreef | Vlaams Brabant | 4.11E-01 |  | 0.05 | NS | GAM | 2008 | 2021 | 10 |
| 623 | Zandstraat | Limburg | 3.41E-02 | 2.50E-02 | 0.04 | I | GAM | 1997 | 2022 | 12 |
| 632 | Zeeweg (Sint-Andries) | West-Vlaanderen | 2.16E-01 |  | -0.02 | NS | GAM | 2003 | 2022 | 16 |
| 637 | Zink (Munte) | Oost-Vlaanderen | 2.52E-03 | 5.07E-01 | 0.02 | F | GAM | 2002 | 2021 | 19 |
| 3 | Aan De Rodeberg (Engsbergen) | Limburg |  | 1.75E-01 | 0.07 | NS | GLM | 2017 | 2022 | 6 |
| 6 | Abdijstraat | Vlaams Brabant | | 2.25E-01 | 0.26 | NS | GLM | 2017 | 2021 | 6 |
| 8 | Abstraat (Terlanen) | Vlaams Brabant | | 2.35E-05 | -0.23 | D | GLM | 2014 | 2022 | 9 |
| 20 | Averegten - Boonmarkt (Hallaar | Antwerpen | | 8.15E-01 | -0.02 | NS | GLM | 2015 | 2022 | 8 |
| 23 | Bakvoordestraat | West-Vlaanderen | | 8.72E-01 | -0.01 | NS | GLM | 1990 | 1997 | 6 |
| 31 | Baverstraat (Elderen) | Limburg |  | 6.25E-01 | 0.07 | NS | GLM | 1989 | 1994 | 6 |
| 34 | Beernemsteenweg (Wildenburg) | West-Vlaanderen | | 1.32E-18 | -0.61 | D | GLM | 2013 | 2018 | 6 |
| 38 | Bentstraat | Limburg |  | 4.10E-01 | -0.05 | NS | GLM | 2013 | 2022 | 9 |
| 42 | Bergstraat (Tombeek) | Vlaams Brabant | | 7.33E-02 | -0.21 | NS | GLM | 2014 | 2021 | 8 |
| 47 | Beukendreef | Antwerpen | | 9.66E-01 | 0.00 | NS | GLM | 2014 | 2021 | 6 |
| 52 | Bijlokestraat | Oost-Vlaanderen | | 2.08E-04 | 0.29 | I | GLM | 2013 | 2022 | 9 |
| 64 | Bogaardenstraat | Vlaams Brabant | | 6.26E-01 | -0.01 | NS | GLM | 2008 | 2021 | 9 |
| 70 | Boshoek | Antwerpen | | 5.76E-07 | -0.21 | D | GLM | 1995 | 2002 | 8 |
| 71 | Boskapellaan | Oost-Vlaanderen | | 6.39E-02 | -0.43 | NS | GLM | 2016 | 2022 | 7 |
| 73 | Bosstraat (Koersel) | Limburg |  | 1.69E-01 | -0.13 | NS | GLM | 2015 | 2022 | 8 |
| 74 | Bosstraat | West-Vlaanderen | | 4.47E-01 | -0.17 | NS | GLM | 2013 | 2021 | 8 |
| 77 | Boudewijnlaan En Buurt | West-Vlaanderen | | 8.89E-01 | 0.01 | NS | GLM | 2017 | 2022 | 6 |
| 80 | Brandstraat, Bosmansdreef | Oost-Vlaanderen | | 5.37E-02 | -0.26 | NS | GLM | 2017 | 2022 | 6 |
| 81 | Broekstraat (Blaasveld) | Antwerpen | | 2.37E-02 | -0.04 | D | GLM | 1999 | 2022 | 7 |
| 82 | Broekstraat, Fonteinstraat | Antwerpen | | 3.43E-01 | 0.02 | NS | GLM | 1988 | 1997 | 7 |
| 84 | Bruggenhoek | Oost-Vlaanderen | | 4.58E-01 | 0.11 | NS | GLM | 2013 | 2021 | 6 |
| 96 | Cleynhenslaan | Vlaams Brabant | | 2.22E-02 | -0.35 | D | GLM | 2011 | 2017 | 6 |
| 98 | Daalbroekstraat (Rekem) | Limburg |  | 4.39E-03 | -0.19 | D | GLM | 2013 | 2021 | 9 |
| 101 | De 3 Bruggen | Antwerpen | | 6.66E-01 | 0.15 | NS | GLM | 2017 | 2022 | 6 |
| 102 | De Bunt | Oost-Vlaanderen | | 2.47E-04 | -0.12 | D | GLM | 2006 | 2022 | 9 |
| 103 | De Hoef | Vlaams Brabant | | 2.20E-01 | -0.64 | NS | GLM | 2013 | 2015 | 6 |
| 108 | Diepstraat (Loppem) | West-Vlaanderen | | 2.34E-01 | -0.05 | NS | GLM | 1988 | 2001 | 8 |
| 110 | Dompels | Oost-Vlaanderen | | 6.66E-01 | 0.04 | NS | GLM | 2015 | 2021 | 7 |
| 111 | Donderheide | Antwerpen | | 2.39E-01 | 0.15 | NS | GLM | 2013 | 2022 | 8 |
| 116 | Drengel | Antwerpen | | 4.55E-02 | -0.14 | D | GLM | 2004 | 2022 | 7 |
| 128 | Edmond Ronsestraat (Oostakker) | Oost-Vlaanderen | | 2.26E-01 | 0.28 | NS | GLM | 2013 | 2018 | 6 |
| 130 | Eekhofstraat | West-Vlaanderen | | 6.66E-01 | 0.02 | NS | GLM | 2015 | 2022 | 8 |
| 132 | Eichemstraat (Eichem) | Oost-Vlaanderen | | 3.24E-01 | -0.04 | NS | GLM | 2009 | 2022 | 6 |
| 143 | Engelbamp (Nieuwenhoven) | Limburg |  | 1.19E-06 | -0.05 | NS | GLM | 1988 | 2022 | 6 |
| 145 | Europawijk | Antwerpen | | 8.89E-01 | 0.01 | NS | GLM | 2003 | 2021 | 7 |
| 149 | Ezelstraat | West-Vlaanderen | | 3.63E-02 | -0.16 | D | GLM | 2011 | 2021 | 9 |
| 155 | Fonteinstraat (Blaasveld) | Antwerpen | | 7.28E-01 | -0.02 | NS | GLM | 2007 | 2022 | 6 |
| 158 | Frans Verbeekstraat (Overijse) | Vlaams Brabant | | 4.69E-07 | -0.37 | D | GLM | 2014 | 2022 | 8 |
| 170 | Gistelstraat | West-Vlaanderen | | 1.69E-01 | -0.12 | NS | GLM | 2011 | 2021 | 9 |
| 172 | Goorboslei | Antwerpen | | 6.66E-01 | -0.06 | NS | GLM | 2016 | 2022 | 6 |
| 173 | Goorstraat (Oelegem) | Antwerpen | | 4.12E-01 | -0.03 | NS | GLM | 2012 | 2018 | 6 |
| 176 | Gotegemstraat | Oost-Vlaanderen | | 5.60E-01 | 0.06 | NS | GLM | 2016 | 2022 | 7 |
| 177 | Groenboomgaardstraat | West-Vlaanderen | | 1.38E-05 | -0.33 | D | GLM | 2015 | 2022 | 8 |
| 178 | Groenenhoek | Antwerpen | | 1.19E-06 | -0.21 | D | GLM | 2006 | 2017 | 8 |
| 180 | Groot Westhof (Nieuwkerke) | West-Vlaanderen | | 5.80E-12 | -0.44 | D | GLM | 1981 | 1988 | 6 |
| 187 | Hagaard (Overijse) | Vlaams Brabant | | 3.16E-03 | -0.64 | D | GLM | 2017 | 2022 | 6 |
| 191 | Hauwerzele | Oost-Vlaanderen | | 2.43E-03 | -0.31 | D | GLM | 2014 | 2022 | 9 |
| 193 | Heidestraat (Zemst) | Vlaams Brabant | | 1.58E-01 | -0.10 | NS | GLM | 2017 | 2022 | 6 |
| 195 | Heidestraat - Zuid | Antwerpen | | 1.64E-02 | 0.38 | I | GLM | 2014 | 2021 | 8 |
| 197 | Heidestraat | Vlaams Brabant | | 6.58E-05 | 0.33 | I | GLM | 2011 | 2017 | 7 |
| 198 | Heiken (O.L.V.Waver) | Antwerpen | | 2.20E-01 | -0.08 | NS | GLM | 2014 | 2022 | 8 |
| 210 | Hoek Ter Hulst (Moortsele) | Oost-Vlaanderen | | 3.18E-01 | -0.03 | NS | GLM | 2013 | 2022 | 6 |
| 213 | Hoevendijk | Antwerpen | | 2.52E-02 | -0.10 | D | GLM | 2014 | 2022 | 9 |
| 221 | Hollebeek En Schauselhoekstraa | Oost-Vlaanderen | | 5.23E-03 | -0.25 | D | GLM | 2012 | 2019 | 8 |
| 223 | Holsteenweg | Limburg |  | 9.37E-07 | 0.33 | I | GLM | 2014 | 2022 | 8 |
| 224 | Hommelhofstraat | West-Vlaanderen | | 1.85E-01 | -0.07 | NS | GLM | 2015 | 2022 | 8 |
| 233 | Houtstraat (Olsene) | Oost-Vlaanderen | | 7.81E-01 | 0.05 | NS | GLM | 2013 | 2018 | 6 |
| 234 | Huybergsebaan | Antwerpen | | 3.88E-10 | -0.32 | D | GLM | 1999 | 2018 | 9 |
| 235 | Ijshoutestraat | Oost-Vlaanderen | | 1.69E-10 | -0.67 | D | GLM | 2012 | 2019 | 6 |
| 243 | Karperstraat | West-Vlaanderen | | 4.62E-01 | -0.08 | NS | GLM | 2011 | 2018 | 8 |
| 249 | Kasteelstraat (Dikkelvenne) | Oost-Vlaanderen | | 2.52E-02 | -0.28 | D | GLM | 2011 | 2018 | 7 |
| 261 | Kerkstraat (Tielrode) | Oost-Vlaanderen | | 4.38E-02 | 0.09 | I | GLM | 2008 | 2019 | 9 |
| 266 | Kievitstraat | Antwerpen | | 6.78E-01 | -0.03 | NS | GLM | 2015 | 2022 | 8 |
| 270 | Klare Grachtstraat | West-Vlaanderen | | 1.61E-02 | -0.29 | D | GLM | 2011 | 2019 | 9 |
| 274 | Kluisstraat | Antwerpen | | 1.24E-01 | -0.05 | NS | GLM | 2001 | 2016 | 8 |
| 278 | Knodbaan (Oelegem) | Antwerpen | | 8.89E-01 | 0.01 | NS | GLM | 2011 | 2018 | 7 |
| 285 | Koolskampstraat | West-Vlaanderen | | 1.76E-08 | -0.27 | D | GLM | 2014 | 2021 | 8 |
| 289 | Koutergoedstraat (Oostakker) | Oost-Vlaanderen | | 1.44E-08 | -0.47 | D | GLM | 2016 | 2022 | 6 |
| 290 | Kouterstraat (Overijse) | Vlaams Brabant | | 2.52E-01 | -0.12 | NS | GLM | 2014 | 2022 | 8 |
| 292 | Kraaibornstraat (Lauw) | Limburg |  | 1.01E-01 | -0.19 | NS | GLM | 2014 | 2021 | 8 |
| 296 | Kruiskerkestraat | West-Vlaanderen | | 3.49E-07 | -0.67 | D | GLM | 2011 | 2021 | 7 |
| 299 | Kuikenstraat | Antwerpen | | 6.66E-01 | -0.07 | NS | GLM | 2013 | 2022 | 9 |
| 302 | Lanestraat (Tombeek) | Vlaams Brabant | | 1.95E-08 | -0.15 | D | GLM | 2014 | 2022 | 9 |
| 304 | Lange Maat, Meerstraat | Oost-Vlaanderen | | 6.38E-01 | 0.05 | NS | GLM | 2014 | 2022 | 8 |
| 310 | Legeweg | West-Vlaanderen | | 1.07E-02 | -0.24 | D | GLM | 1990 | 2001 | 8 |
| 311 | Lembergestraat (Landskouter) | Oost-Vlaanderen | | 3.68E-08 | -0.12 | D | GLM | 2011 | 2022 | 8 |
| 322 | Lindebornstraat (Elderen) | Limburg |  | 9.87E-06 | -0.16 | D | GLM | 1989 | 2006 | 9 |
| 326 | Lotenhullestraat | Oost-Vlaanderen | | 4.54E-05 | 0.10 | I | GLM | 2004 | 2022 | 7 |
| 332 | M. Noëstraat | Vlaams Brabant | | 1.63E-08 | 0.17 | I | GLM | 2008 | 2021 | 9 |
| 342 | Meersakkerstraat | Oost-Vlaanderen | | 2.34E-01 | 0.11 | NS | GLM | 2015 | 2022 | 8 |
| 343 | Meersstraat, Scheutlagestraat | Oost-Vlaanderen | | 1.68E-12 | -0.51 | D | GLM | 2015 | 2022 | 8 |
| 356 | Milleniumvijver (Elewijt) | Vlaams Brabant | | 5.16E-02 | 0.12 | NS | GLM | 2012 | 2022 | 9 |
| 375 | Nachtegalenstraat | Vlaams Brabant | | 1.79E-01 | 0.09 | NS | GLM | 2015 | 2021 | 6 |
| 379 | Neremweg | Limburg |  | 1.88E-01 | -0.13 | NS | GLM | 2015 | 2022 | 8 |
| 383 | Nieuwstraat | Vlaams Brabant | | 1.28E-29 | -0.18 | D | GLM | 1991 | 2021 | 7 |
| 385 | Ninoofsesteenweg | Oost-Vlaanderen | | 6.46E-06 | 0.12 | I | GLM | 2012 | 2021 | 9 |
| 399 | Oude Gentweg | Oost-Vlaanderen | | 2.55E-11 | 0.13 | I | GLM | 2003 | 2016 | 8 |
| 400 | Oude Lichterveldsestraat | West-Vlaanderen | | 1.58E-01 | -0.29 | NS | GLM | 2015 | 2020 | 7 |
| 402 | Oude Maria Lindestraat | West-Vlaanderen | | 2.66E-02 | 0.38 | I | GLM | 2013 | 2021 | 7 |
| 421 | Parkstraat | West-Vlaanderen | | 1.45E-09 | -0.63 | D | GLM | 2011 | 2017 | 6 |
| 424 | Pater Penninckxstraat | Vlaams Brabant | | 4.38E-02 | 0.12 | I | GLM | 2015 | 2022 | 8 |
| 440 | Populierenlaan | Vlaams Brabant | | 6.66E-01 | -0.07 | NS | GLM | 2014 | 2021 | 6 |
| 452 | Remerstraat | Vlaams Brabant | | 5.55E-04 | -0.28 | D | GLM | 2011 | 2016 | 6 |
| 453 | Reppelerweg (Grote Brogel) | Limburg |  | 8.54E-02 | -0.10 | NS | GLM | 2005 | 2022 | 7 |
| 454 | Retiesebaan | Antwerpen | | 3.20E-01 | -0.12 | NS | GLM | 2015 | 2022 | 6 |
| 459 | Rijkegemkouter | West-Vlaanderen | | 3.26E-01 | -0.09 | NS | GLM | 2011 | 2021 | 8 |
| 469 | Rondrit In Gemeente | West-Vlaanderen | | 2.57E-01 | -0.20 | NS | GLM | 2013 | 2022 | 6 |
| 471 | Rosendaelweg | Antwerpen | | 4.21E-02 | -0.15 | D | GLM | 2013 | 2022 | 8 |
| 478 | Salphensebaan | Antwerpen | | 9.53E-15 | -0.42 | D | GLM | 2016 | 2022 | 7 |
| 481 | Schalmeidreef | Antwerpen | | 9.66E-01 | 0.00 | NS | GLM | 2008 | 2016 | 7 |
| 486 | Scheldebroeken (Zele-Dijk) | Oost-Vlaanderen | | 9.45E-01 | 0.00 | NS | GLM | 2000 | 2014 | 9 |
| 487 | Scheldeveldstraat | Oost-Vlaanderen | | 6.36E-01 | -0.09 | NS | GLM | 2014 | 2020 | 7 |
| 495 | Schoterheide | Limburg |  | 5.37E-02 | -0.11 | NS | GLM | 2014 | 2022 | 9 |
| 504 | Sint-Annalaan - Heuken | Vlaams Brabant | | 4.77E-02 | 0.04 | I | GLM | 2008 | 2022 | 7 |
| 507 | Sint-Pauluslaan (Huize Walden) | Antwerpen | | 1.18E-01 | -0.12 | NS | GLM | 2014 | 2022 | 9 |
| 521 | Spinele | Oost-Vlaanderen | | 2.01E-01 | -0.14 | NS | GLM | 2014 | 2021 | 7 |
| 526 | Spoorwegstraat | West-Vlaanderen | | 7.53E-01 | -0.05 | NS | GLM | 2013 | 2022 | 9 |
| 535 | Steenstraat (Westende) | West-Vlaanderen | | 5.44E-19 | -0.89 | D | GLM | 2011 | 2018 | 8 |
| 547 | Terluchtestraat (Ruddervoorde) | West-Vlaanderen | | 3.20E-01 | -0.17 | NS | GLM | 1998 | 2003 | 6 |
| 549 | Tillegembos (Sint Michiels) | West-Vlaanderen | | 3.88E-10 | -0.31 | D | GLM | 2006 | 2016 | 7 |
| 550 | Tinnenpotstraat-Lijsterstraat | West-Vlaanderen | | 2.29E-03 | -0.32 | D | GLM | 2013 | 2021 | 9 |
| 553 | Torrestraat (Machelen) | Oost-Vlaanderen | | 7.85E-01 | 0.03 | NS | GLM | 2000 | 2007 | 7 |
| 555 | Tulpenlaan | West-Vlaanderen | | 2.42E-03 | -0.22 | D | GLM | 2015 | 2022 | 8 |
| 558 | Varenstraat | Antwerpen | | 6.27E-01 | 0.12 | NS | GLM | 2014 | 2022 | 7 |
| 559 | Varestraat | Antwerpen | | 1.83E-03 | -0.18 | D | GLM | 1996 | 2004 | 6 |
| 565 | Vijfstraten | Vlaams Brabant | | 8.89E-01 | -0.01 | NS | GLM | 2011 | 2019 | 9 |
| 579 | Vrijbosstraat | West-Vlaanderen | | 6.78E-01 | -0.03 | NS | GLM | 2014 | 2019 | 6 |
| 584 | Waaienburgseweg (Roesbrugge) | West-Vlaanderen | | 1.75E-01 | -0.14 | NS | GLM | 2006 | 2015 | 8 |
| 593 | Waterstraat | Antwerpen | | 8.59E-01 | 0.02 | NS | GLM | 2007 | 2021 | 6 |
| 620 | Zand | Antwerpen | | 1.30E-01 | 0.25 | NS | GLM | 2017 | 2022 | 6 |
| 624 | Zandstraat, Drengel | Antwerpen | | 3.20E-04 | -0.13 | D | GLM | 2012 | 2021 | 9 |
| 626 | Zandstraat (Geel) | Antwerpen | | 7.81E-01 | 0.01 | NS | GLM | 1998 | 2022 | 6 |
| 628 | Zandstraat (Sint-Katelijne-Waver) | Antwerpen | | 3.12E-01 | -0.18 | NS | GLM | 2016 | 2021 | 6 |

Appendix Fig. S2:

Using the trend for Waasmunsterbaan, Lokeren (site id: 585), we visually illustrate the trend inference using GAMs. We show the smoother (green line), counts of the yearly surveys (black dots) and the 95% CI (grey zone) as a log transformed values.

The first step was performing an omnibus test, which tested whether the smoother is significantly different from a flat line at any point in time. Since we centered all values, this meant that we assessed whether the smoother is different from zero at any point in time. In this example, we see that the zero-line is not included in the 95% CI over the whole time series of the smoother. This means that there is a trend and that this is not a non-significant population. In this research we consider the Begin vs End contrast as the best measure for overall change. Therefore, the second step compares the begin and end point (indicated in green squares). We determined if the difference (end – begin) is significantly different from zero. Visually this means that the end point is not included in the 95% CI of the begin point. When this is significantly different from zero, then there is a directional trend depending on the sign of the difference: ‘increase’ when positive and ‘decline’ when negative. Otherwise there is a non-monotonic trend, which we would label as ‘flexible’. In this case there is a directional trend with a negative difference and thus a declining population. Remark that the level of decline can also be computed as such: (exp(contrast) – 1) * 100)

Because we performed more than 100 GAMs, we corrected the p-values for multiple testing using the stage-wise analysis.


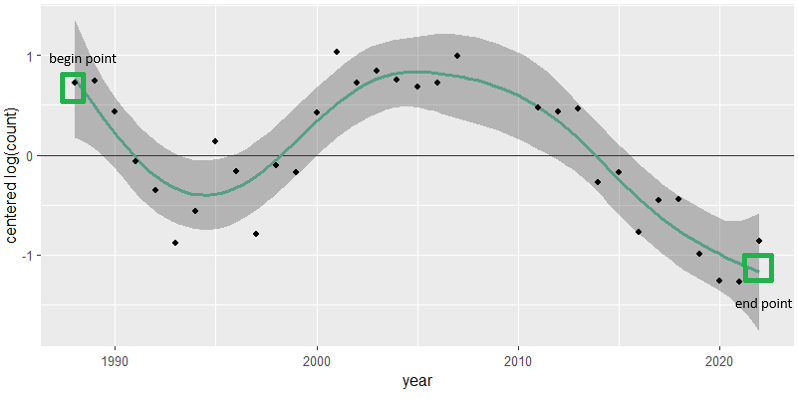


Appendix Table S3:

This Table shows which initial land use components extracted from the BWK-BBK hybrid map make up the final land use components used in the regression model and gives a short description of what they contain.

| Initial Component | Final Component | Description |
| --- | --- | --- |
| Arable land  Agricultural Grass & Shrubs | Agricultural land | Agricultural area, ranging from bare arable fields to meadows and pastures. |
| Woodland | Woodland | Forested area |
| Buildings  Roads  Covered  Uncovered  Grass and Shrubs | Urbanized area | Build-up area and gardens and roadsides, towns, villages, industrial areas,… |
| Water | Water | Bodies of water, ranging from ponds to rivers, lakes and channels. |
| BWK Grasslands | BWK Grasslands | Grasslands indicated on the Biological valuation map that are not classified as agricultural Grass & Shrubs or Water on the BBK. |
| Railways | Railways | Railways |

Appendix Fig. S4:

Lineplots detailing the change the proportion of different land-use components on the y-axis and how they change between 2012 and 2021 (x-axis). The colours represent the different labels of Common Toad trend values (D = decrease, F = flexible, I = increasing, NS = non-significant).

100m Scale


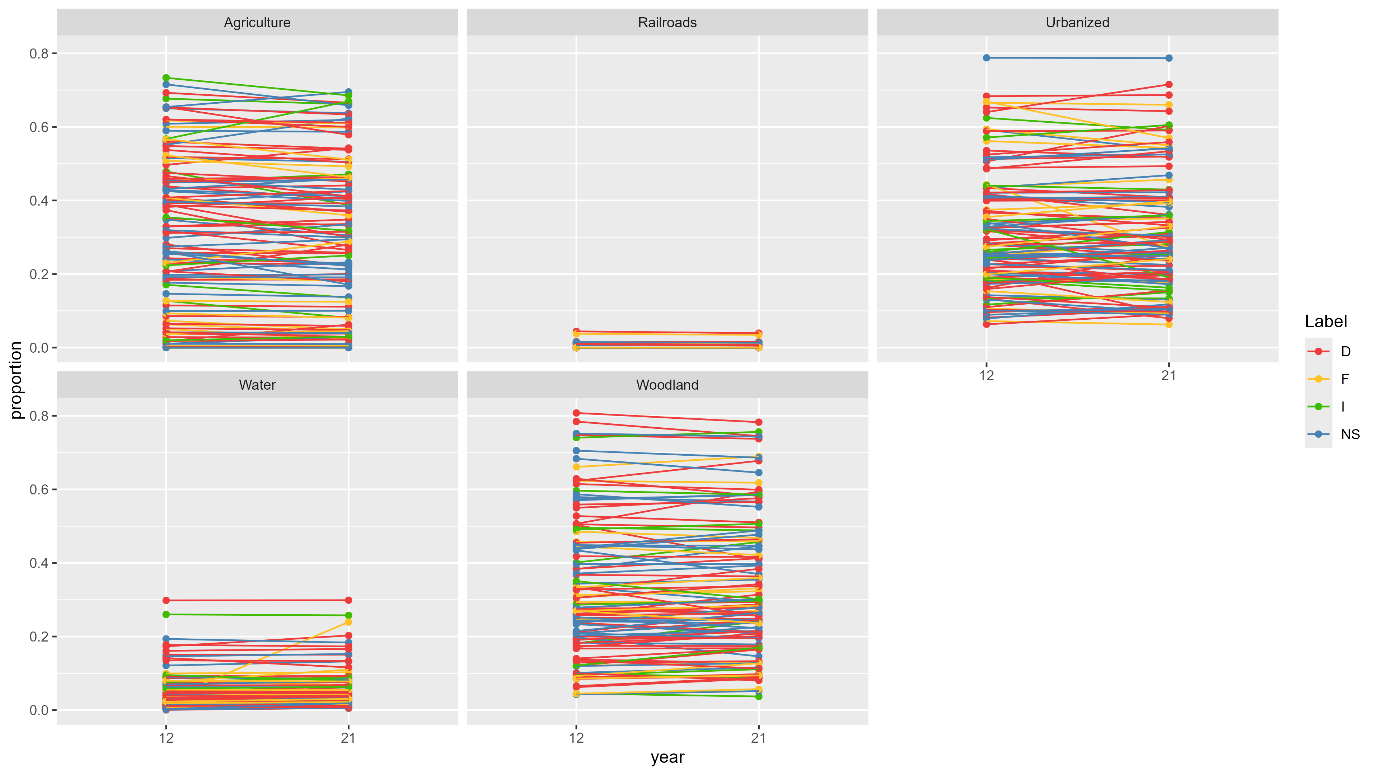

500m Scale


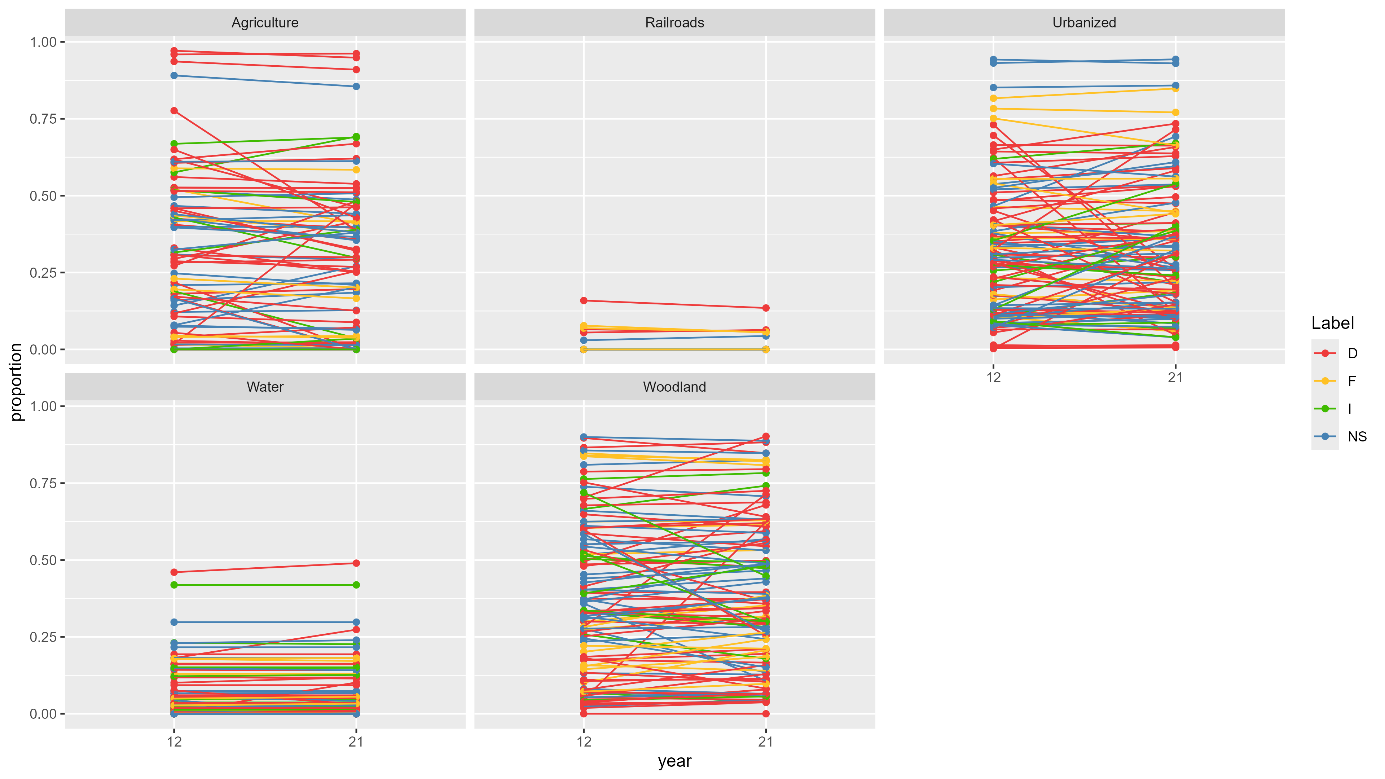


1000m Scale


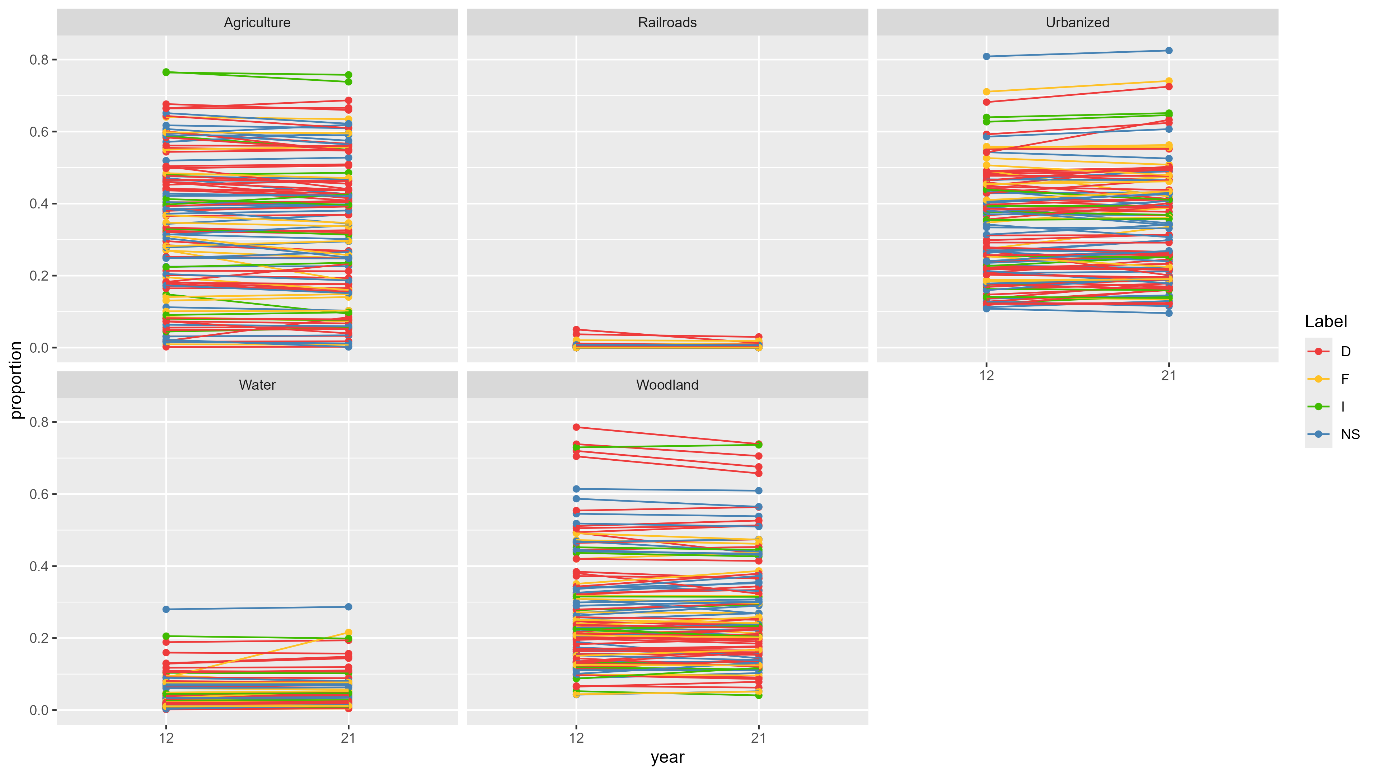


Appendix information S5:

The details of this modification can be seen in the following formula, for brevity we assume three different components instead of six:

We know that:

$$X_{1}+X_{2}+X_{3}=1 \leftrightarrow X_{3}=1-(X_{1}+X_{2})$$

So, within a standard linear model this would result in the following equation:

$$Y_{i}=\beta_{0}+\beta_{1}X_{1,i}+\beta_{2}X_{2,i}+\beta_{3}X_{3,i}+\epsilon_{i}$$

$$\leftrightarrow Y_{i}=\beta_{0}+\beta_{1}X_{1,i}+\beta_{2}X_{2,i}+\beta_{3}(1-(X_{2,i}+X_{1,i}))+\epsilon_{i}$$

$$\leftrightarrow Y_{i}={(\beta}_{0}+\beta_{3})+{(\beta}_{1}-\beta_{3})X_{1,i}+(\beta_{2}-\beta_{3})X_{2,i}+ \epsilon_{i}$$

In this case we used agricultural land as reference class, but this can be any land use component that is used as predictor variable.

Appendix Table S6:

The Eigenvalues and the proportion of explained variation of the different PC axes for the PCA ordinations of land use proportions at 100, 500 and 1000 m.

PCA 100 m

|  | PC1 | PC2 | PC3 | PC4 | PC5 | PC6 | PC7 | PC8 | PC9 | PC10 |
| --- | --- | --- | --- | --- | --- | --- | --- | --- | --- | --- |
| Eigenvalue | 2.52 | 1.65 | 1.21 | 1.11 | 1.04 | 0.99 | 0.82 | 0.63 | 0.55 | 0.47 |
| Proportion Explained | 0.23 | 0.15 | 0.11 | 0.10 | 0.095 | 0.090 | 0.074 | 0.057 | 0.050 | 0.043 |
| Cumulative Proportion | 0.23 | 0.38 | 0.49 | 0.59 | 0.68 | 0.77 | 0.85 | 0.91 | 0.96 | 1.00 |

PCA 500 m

|  | PC1 | PC2 | PC3 | PC4 | PC5 | PC6 | PC7 | PC8 | PC9 | PC10 |
| --- | --- | --- | --- | --- | --- | --- | --- | --- | --- | --- |
| Eigenvalue | 4.06 | 2.06 | 1.11 | 1.01 | 0.89 | 0.58 | 0.50 | 0.37 | 0.29 | 0.14 |
| Proportion Explained | 0.37 | 0.19 | 0.10 | 0.092 | 0.081 | 0.052 | 0.045 | 0.034 | 0.027 | 0.013 |
| Cumulative Proportion | 0.37 | 0.56 | 0.66 | 0.75 | 0.83 | 0.88 | 0.93 | 0.96 | 0.99 | 1.00 |

PCA 1000 m

|  | PC1 | PC2 | PC3 | PC4 | PC5 | PC6 | PC7 | PC8 | PC9 | PC10 |
| --- | --- | --- | --- | --- | --- | --- | --- | --- | --- | --- |
| Eigenvalue | 4.48 | 2.09 | 1.09 | 0.98 | 0.84 | 0.52 | 0.40 | 0.33 | 0.20 | 0.080 |
| Proportion Explained | 0.41 | 0.19 | 0.099 | 0.089 | 0.076 | 0.047 | 0.036 | 0.030 | 0.018 | 0.0073 |
| Cumulative Proportion | 0.41 | 0.60 | 0.70 | 0.79 | 0.86 | 0.91 | 0.94 | 0.97 | 0.99 | 1.00 |

Appendix Fig. S7:

PCA ordination biplot for the 100 m and 1000 m buffer scale. Different colours detail different labels of toad population trends. Marginal density graphs describe the density of different populations corresponding to different trend labels.


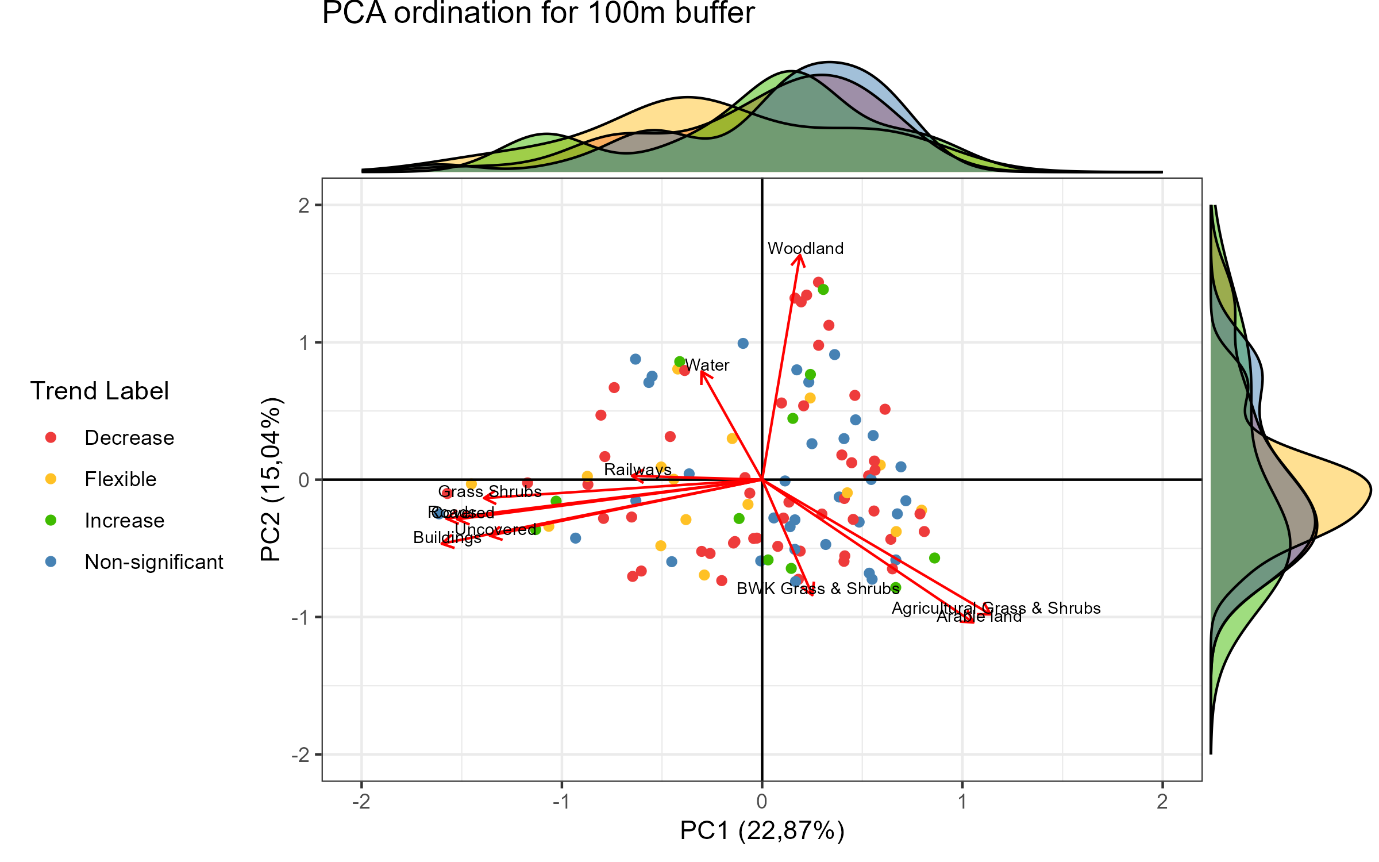

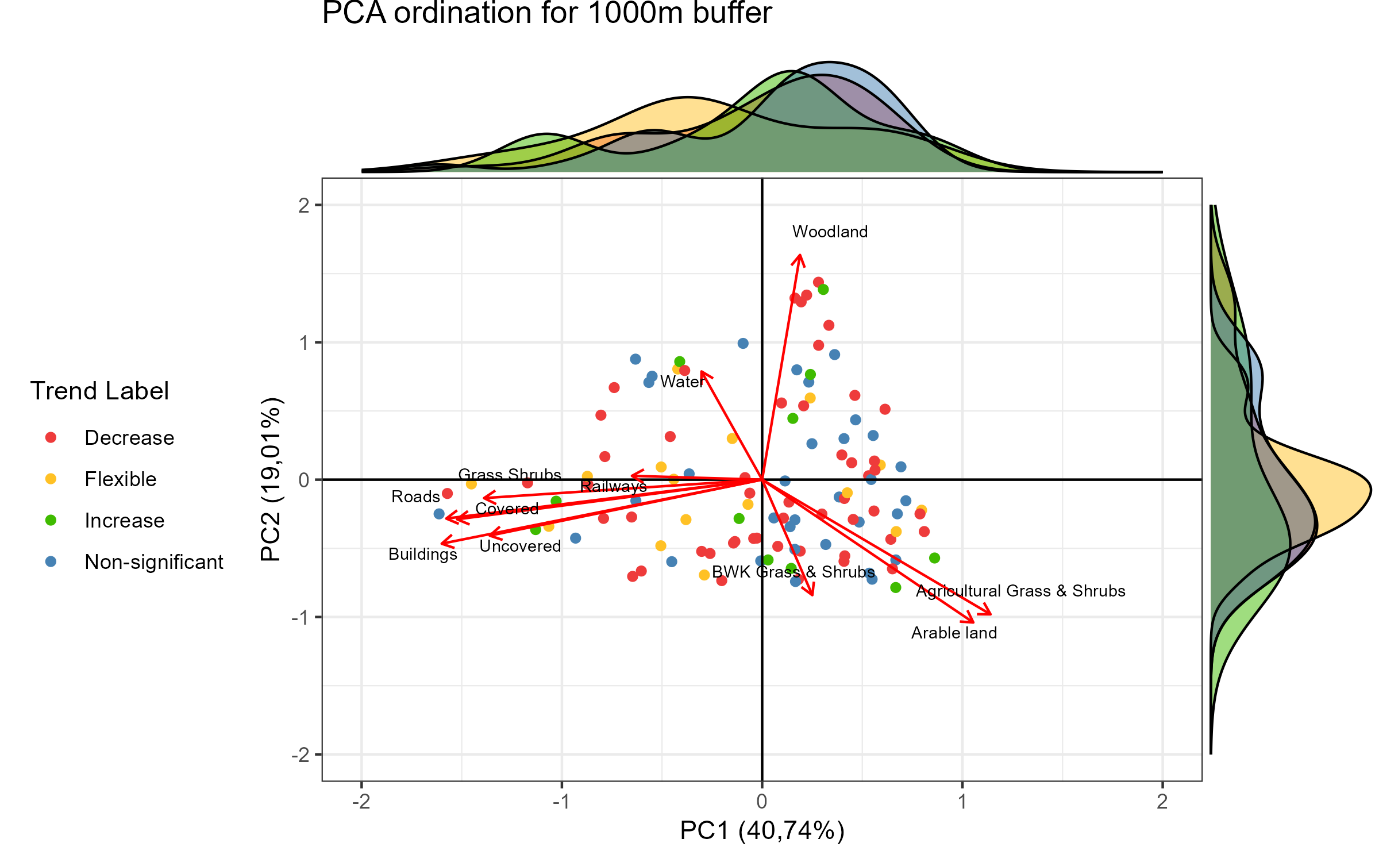

Supplement: Supplementary file 1 — Data S1: ece373653‐sup‐0001‐Supinfo.docx. [file ECE3-16-e73653-s001.docx]
